# Aryl hydrocarbon receptor is a proviral host factor and a candidate pan-SARS-CoV-2 therapeutic target

**DOI:** 10.1126/sciadv.adf0211

**Published:** 2023-05-31

**Authors:** Jiandong Shi, Tingfu Du, Junbin Wang, Cong Tang, Mengyue Lei, Wenhai Yu, Yun Yang, Ying Ma, Pu Huang, Hongli Chen, Xu Wang, Jing Sun, Haixuan Wang, Yong Zhang, Fangyu Luo, Qing Huang, Bai Li, Shuaiyao Lu, Yunzhang Hu, Xiaozhong Peng

**Affiliations:** ^1^ National Kunming High-level Biosafety Primate Research Center, Institute of Medical Biology, Chinese Academy of Medical Sciences and Peking Union Medical College, Beijing, China.; ^2^ State Key Laboratory of Medical Molecular Biology, Department of Molecular Biology and Biochemistry, Institute of Basic Medical Sciences, Medical Primate Research Center, Neuroscience Center, Chinese Academy of Medical Sciences, School of Basic Medicine, Peking Union Medical College, Beijing China.; ^3^ Institute of Laboratory Animal Sciences, Chinese Academy of Medical Sciences and Peking Union Medical College, Beijing China.

## Abstract

The emergence of a series of SARS-CoV-2 variants has necessitated the search for broad-spectrum antiviral targets. The aryl hydrocarbon receptor (AhR) senses tryptophan metabolites and is an immune regulator. However, the role of AhR in SARS-CoV-2 infection and whether AhR can be used as the target of antiviral therapy against SARS-CoV-2 and its variants are yet unclear. Here, we show that infection with SARS-CoV-2 activates AhR signaling and facilitates viral replication by interfering with IFN-I–driven antiviral immunity and up-regulating ACE2 receptor expression. The pharmacological AhR blockade or AhR knockout reduces SARS-CoV-2 and its variants’ replication in vitro. Drug targeting of AhR with AhR antagonists markedly reduced SARS-CoV-2 and its variants’ replication in vivo and ameliorated lung inflammation caused by SARS-CoV-2 infection in hamsters. Overall, AhR was a SARS-CoV-2 proviral host factor and a candidate host-directed broad-spectrum target for antiviral therapy against SARS-CoV-2 and its variants, including Delta and Omicron, and potentially other variants in the future.

## INTRODUCTION

Coronavirus disease 2019 (COVID-19) caused by severe acute respiratory syndrome coronavirus 2 (SARS-CoV-2) is a emerging infectious disease that has spread worldwide, resulting in a global pandemic (*1*). The pandemic has caused 6.25 million deaths and remains a severe challenge to global public health security (*2*). SARS-CoV-2 causes extensive damage in various organ systems mediated by the host immune response. The severity of the disease ranges from asymptomatic infection to severe multiple organ failure (*3*). To develop prevention and treatment strategies, several studies have been performed to understand the host-virus interaction and viral pathogenesis. However, COVID-19 is still a new disease, and its complete pathogenesis remains to be clarified, especially the key cellular and molecular events in the early stage of virus infection have not been deciphered. In addition, the effective and specific antiviral drugs against SARS-CoV-2 are still limited. Therefore, finding candidate targets for the treatment and prevention of SARS-CoV-2 infection is an urgent requisite.

In addition to viral angiotensin-converting enzyme 2 (ACE2) and transmembrane protease serine 2 (TMPRSS2) receptors, SARS-CoV-2 also relies on host proteins to promote its replication. The identification of host proviral factors crucial to SARS-CoV-2 replication would reveal new therapeutic targets and develop new host targeted antiviral strategies. Recent studies have identified some host proviral factors of SARS-CoV-2 replication, including a disintegrin and metalloprotease 10 (ADAM10), a disintegrin and metalloprotease 17 (ADAM17) (*4*), transmembrane protein 41B (TMEM41B) (*5*, *6*), GATA binding protein 6 (GATA6) (*7*), and bromodomain-containing protein 2 (BRD2) (*8*). Moreover, the genes and pathways of viral action, including High mobility group protein B1 (HMGB1) and the SWItch/Sucrose Nonfermentable (SWI/SNF) chromatin remodeling complex (*9*), were also identified. These host proteins and pathways are crucial to the life cycle of virus, i.e., when they enter the human cells or establish viral replication.

The global spread of the virus has evolved many new variants under the pressure of host immune selection (*10*). The emergence of a series of SARS-CoV-2 variants has caused a huge impact on the vaccine-regulated immune barriers throughout the world. The new mutant strains escape the immunity and reduce the protective effect of the original vaccine (*11*), leading to drug resistance to the virus. Therefore, compared to the classical methods targeting viral components, the antiviral therapy strategy targeting host factors provides a potential alternative approach. The antiviral strategies targeting host factors have two advantages: (i) They have broad-spectrum antiviral properties because they target factors required for virus replication, and (ii) The risk of producing immune escape strains and drug-resistant mutants is greatly reduced (*12*). However, the broad-spectrum antiviral targets for different variants of SARS-CoV-2 are limited, and additional candidates need to be investigated.

The aryl hydrocarbon receptor (AhR) is a ligand-activated transcription factor and a nuclear receptor. Similar to other nuclear receptors, the activation of AhR is ligand-driven. In the absence of ligands, AhR is localized in the cytoplasm as a part of the molecular chaperone complex (*13*). After activated by the ligand, it enters the nucleus and forms heterodimers with AhR nuclear translocator and specifically binds to AhR reaction elements. This interaction between AhR and its ligand activates the expression of a series of genes downstream of AhR, such as cytochrome P4501A1 (*CYP1A1*) (*13*). The AhR is widely expressed in innate and adaptive immune cells. AhR has a variety of physiological functions, including the regulation of immune and inflammatory processes and environmental responses (*13*, *14*). The activation of indoleamine 2,3-dioxygenase 1 (IDO1) causes immune cells to release kynurenine (Kyn), a tryptophan (Trp) metabolite, and an endogenous ligand that activates AhR, which interferes with protective immunity (*14*–*17*). Recent studies have confirmed that AhR regulates viral infection and replication through various pathways, thereby providing a survival advantage to many viruses. Among these, Zika virus infection activates AHR, which limits the production of type I interferon (IFN-I) (*18*). AhR activation suppresses the initiation of influenza virus-specific CD8^+^ T cells in the lung (*19*). Liu *et al.* (*20*) reported that AhR drives the hypersecretion of lung mucins after SARS-CoV-2 infection. In addition, murine coronavirus infection activates AhR, thus prompting proviral 2,3,7,8-tetrachlorodibenzo-p-dioxin (TCDD)-inducible poly(adenosine diphosphate–ribose) polymerase expression and regulating cytokines (*21*). Altered Trp metabolism into the Kyn pathway was also observed on the basis of the metabolomic analysis of patients with COVID-19 (*22*). A recent study reported that AhR is activated by infection with SARS-CoV-2 to promote viral replication, suggesting the potentially novel role for AhR modulation as treatment during SARS-CoV-2 infection (*23*). These cumulative results raise the questions whether AhR plays a role as a proviral host factor in SARS-CoV-2 replication and whether it is feasible for AhR to be the target of broad-spectrum antiviral therapy against SARS-CoV-2 and its variants.

The present study investigated the role of AhR in SARS-CoV-2 infection and evaluated the potential of AhR as a target for host-directed antiviral therapy. Here, our study provided the first in vivo biological validation of AhR inhibition as treatment during SARS-CoV-2 infection. We also showed that infection with SARS-CoV-2 activates AhR signaling and facilitates viral replication by interfering with IFN-I–driven antiviral immunity and up-regulating ACE2 receptor expression. The pharmacological AhR blockade or AhR knockout reduces SARS-CoV-2 and its variants’ replication in vitro. Drug targeting AHR reduces virus replication in hamsters and reverses the pathological damage of lung inflammation caused by virus infection in vivo. In summary, we identified AhR as a SARS-CoV-2 proviral host factor and a candidate host-directed target for antiviral therapy. Together, these findings deemed AhR as a candidate pan-SARS-CoV-2 therapeutic target against SARS-CoV-2 and its variants, including Omicron, and other potential variants in the future.

## RESULTS

### SARS-CoV-2 infection activates AhR signaling in an IFN-IDO-Kyn–dependent pathway

**Fig. 1.**
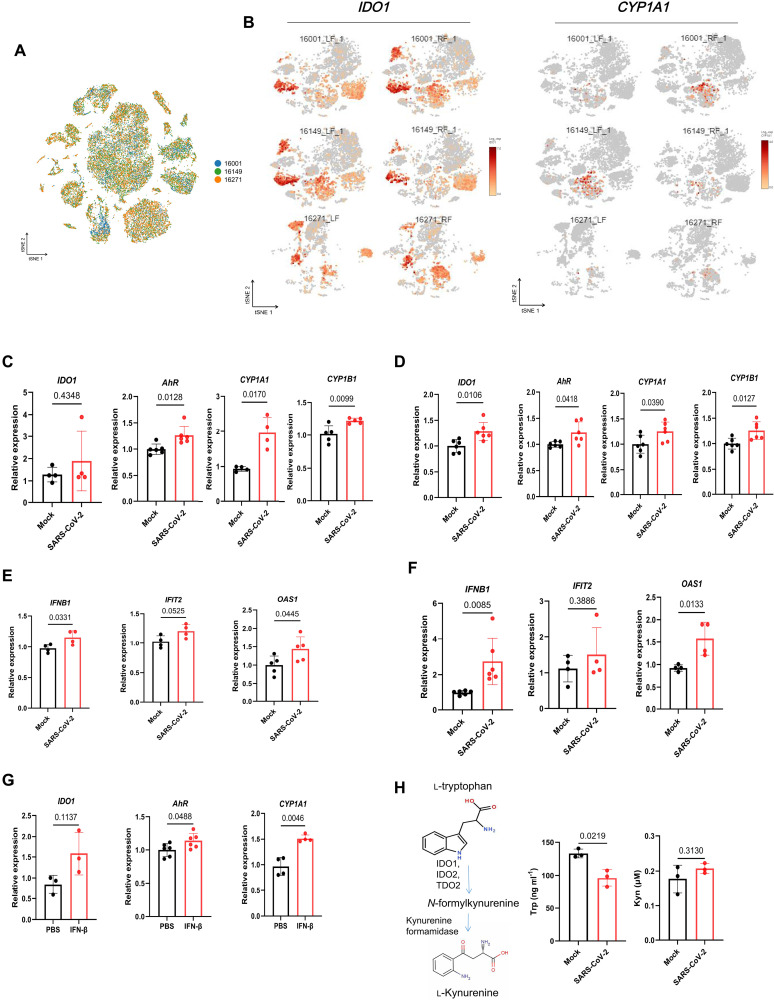
SARS-CoV-2 infection activates AhR signaling in an IFN-IDO-Kyn–dependent pathway. (**A**) Visualization of single cells across different cell types in monkeys. tSNE plots show the cell type annotation of single cells. (**B**) Scatter plot of *IDO1* and *CYP1A1* mRNA expression in lung tissues of macaques infected with SARS-CoV-2. Upstream gene *IDO1* and downstream gene *CYP1A1* mRNA expression of AhR was determined by scRNA-seq. Monkey numbers 16001 and 16149 are SARS-CoV-2–infected monkeys, and monkey number 16271 is SARS-CoV-2–uninfected monkey. (**C**) mRNA expression of genes in the AhR pathway determined by quantitative polymerase chain reaction (qPCR) in H1299 cells at 24 hours after SARS-CoV-2 infection [multiplicity of infection (MOI) of 0.1]. Expression values are relative to mock-infected cells. (**D**) mRNA expression of genes in the AhR pathway determined by qPCR in Calu-3 cells at 24 hours after SARS-CoV-2 infection (MOI of 0.1). Expression values are relative to mock-infected cells. (**E**) Relative expression of IFN-β and ISGs *IFIT2* and *OAS1* in H1299 cells upon SARS-CoV-2 infection (MOI of 0.1). Expression values are relative to mock-infected cells. (**F**) Relative expression of IFN-β and ISGs *IFIT2* and *OAS1* in Calu-3 cells at 24 hours after SARS-CoV-2 infection (MOI of 0.1). Expression values are relative to mock-infected cells. (**G**) mRNA expression of *IDO1*, *AhR*, and *CYP1A1* was determined by qPCR in IFN-β–treated and IFN-β–untreated H1299 cells. (**H**) Enzyme-linked immunosorbent assay (ELISA) quantification of Trp and Kyn in culture supernatants 48 hours after infection of H1299 cells with SARS-CoV-2 (MOI of 0.1).

To determine the effect of SARS-CoV-2 infection on AhR signaling, we performed single-cell RNA sequencing (scRNA-seq) from macaque lung tissue infected with SARS-CoV-2. The lung cell clusters corresponding to endothelial cells, epithelial cells, fibroblasts, gametocytes, and macrophages were identified (Fig. 1A and fig. S1). The scRNA-seq results showed the increased expression of upstream gene *IDO1* and identified the downstream transcriptional targets CYP1A1 and CYP1B1 of AhR in response to SARS-CoV-2 infection (Fig. 1B). Next, we detected the expression of AhR pathway genes *(IDO1*, *AhR*, *CYP1A1*, and *CYP1B1*) in SARS-CoV-2–infected and mock-infected human lung cancer cells (H1299 and Calu-3). In agreement with the scRNA-seq results of macaque lung tissue infected with SARS-CoV-2, we detected an increased expression of IDO1 and AhR and its target genes *CYP1A1* and *CYP1B1* in SARS-CoV-2–infected H1299 and Calu-3 cells (Fig. 1, C and D). Some studies also reported that AhR is activated in cells infected with mouse coronavirus and SARS-CoV-2 (*21*, *23*). Typically, AhR signaling in SARS-CoV-2–infected human lung epithelial cells Calu-3 and COVID-19 patients was observed on the basis of the available gene expression datasets of the viral infection (fig. S2). Obviously, AhR signaling and other various pathways were significantly up-regulated after virus infection. However, AhR signaling was chosen for further studies on the basis of its potential role in promoting SARS-CoV-2 replication.

**Fig. 2.**
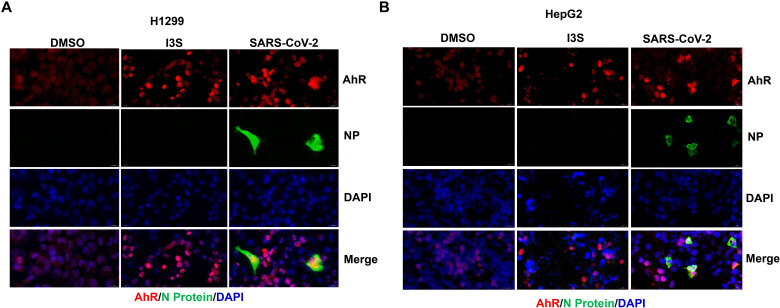
SARS-CoV-2 infection promotes AhR entry into the nucleus. (**A**) H1299 cells and HepG2 cells (**B**) were treated with dimethyl sulfoxide (DMSO) or indoxyl-3-sulfate (I3S) (200 μM) or infected with SARS-CoV-2 (MOI of 0.1) infection for 48 hours. Cells were immunostained with an anti-AhR antibody or anti–SARS-CoV-2 NP antibody and observed under a confocal microscope. Scale bars, 20 μm. Representative images are from three independent experiments. DAPI, 4′,6-diamidino-2-phenylindole.

Next, we studied the molecular pathway of AhR signaling gene expression activated by SARS-CoV-2 infection. Because of the IFN-activated AhR signaling (*24*, *25*), we detected the expression of IFN and IFN-stimulated genes (ISGs), including *IFNB1*, *IFIT1*, and *OAS1*, in SARS-CoV-2–infected and mock-infected H1299 and Calu-3 cells. The results showed that *IFNB1*, *IFIT1*, and *OAS1* were significantly up-regulated in SARS-CoV-2 infection (Fig. 1, E and F). Further results were observed in cells treated with IFN-β accompanied by the increased expression of AhR pathway genes (Fig. 1G), suggesting that IFN-β activates AhR signaling. Because IFNs can activate IDO1 to promote the metabolism of Trp to Kyn (*24*, *25*), we simultaneously detected the changes in Trp and Kyn metabolites in the cell supernatant after SARS-CoV-2 infection. The results showed that the Trp content in the supernatant of virus-infected cells significantly decreased and that the Kyn content increased in a multiplicity of infection (MOI)–dependent manner (Fig. 1H and fig. S3). Moreover, immunofluorescence analysis showed that SARS-CoV-2 infection promotes the nuclear entry of AhR in H1299 and HepG2 cells (Fig. 2). These results indicated that SARS-CoV-2 infection induced IFN production, which further promotes Trp metabolism to produce Kyn. As an endogenous ligand of AhR, Kyn promotes the entry of AhR from the cytoplasm into the nucleus and initiates the expression of AhR transcription target genes. Specifically, IFN-activated signal transducer and activator of transcription 1 up-regulates IDO expression, triggering the Kyn-AhR signaling pathway (*24*, *25*). Similar findings of IFN-activated cytoplasmic transcription factor AhR were also observed in tumor cells (*24*, *25*). Together, these results demonstrated that SARS-CoV-2 infection activates AhR in an IFN-IDO-Kyn–dependent pathway.

### AhR acts as a proviral host factor for SARS-CoV-2 infection

**Fig. 3.**
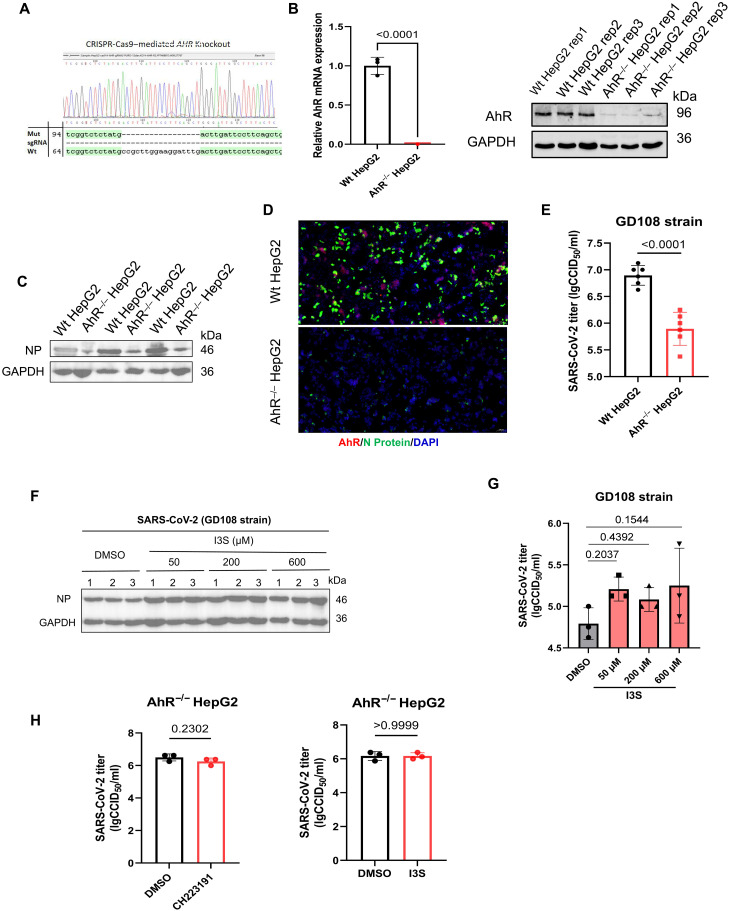
AhR acts as a proviral host factor for SARS-CoV-2 infection. (**A**) AhR^−/−^ HepG2 cell line was generated by CRISPR-Cas9–mediated genome engineering. (**B**) Confirmation of AhR deletion in AhR^−/−^ HepG2 cells at the mRNA and protein levels. GAPDH, glyceraldehyde-3-phosphate dehydrogenase. (**C**) Immunoblot analysis of SARS-CoV-2 NP protein expression in wild-type (Wt) and AhR^−/−^ HepG2 cells. (**D**) Immunofluorescence assay of SARS-CoV-2 NP protein and cellular AhR protein expression in wild-type and AhR^−/−^ HepG2 cells by microscopy (scale bars, 100 μm). (**E**) Viral titer was determined by CCID_50_ in wild-type and AhR^−/−^ HepG2 cells at 24 hours postinfection (hpi) with 0.1 MOI. (**F**) Immunoblot analysis of SARS-CoV-2 NP protein expression in I3S- or DMSO-treated Huh7 cells. Huh7 cells were pretreated with different concentrations of I3S or DMSO and infected with SARS-CoV-2 (MOI of 0. 1) for 24 hours (**G**) Viral titer was determined by CCID_50_ in I3S- or DMSO-treated Huh7 cells. Data from at least three independent experiments (means ± SD). *P* values were determined using a two-tailed, unpaired Student’s *t* test. (**H**) Viral titer was determined by CCID_50_ in AhR^−/−^ HepG2 cells treated with CH223191 or I3S at 24 hpi with MOI of 0.1.

Next, to determine the necessity of AhR in SARS-CoV-2 infection, we constructed an AhR knockout HepG2 cell line (AhR^−/−^ HepG2) by CRISPR-Cas9 (Fig. 3A). The deletion of AhR expression was verified by real-time quantitative polymerase chain reaction (RT-qPCR) and Western blotting (Fig. 3B). The wild-type HepG2 and AhR^−/−^ HepG2 cells were infected with SARS-CoV-2. At 24 hours postinfection (hpi), the level of N protein of SARS-CoV-2 and viral titer was measured. The results showed that the expression level and fluorescence intensity of N protein of SARS-CoV-2 in AhR^−/−^ HepG2 cells was significantly lower than that in the wild-type HepG2 cells (Fig. 3, C and D). Notably, the viral titer decreased significantly in AhR^−/−^ HepG2 cells compared to the wild-type HepG2 cells (Fig. 3E), as determined by the median tissue culture infective dose (TCID_50_) assay. These results demonstrated that AhR is essential for SARS-CoV-2 infection. Furthermore, AhR activation with AhR agonist indoxyl-3-sulfate (I3S) (*26*) increased the N protein level and SARS-CoV-2 viral tier (Fig. 3, F and G). We further confirmed that the AhR agonist and antagonist have no effect on SARS-CoV-2 replication in AhR^−/−^ HepG2 cells, as showed by viral titer (Fig. 3H). Together, these results indicated that AhR is essential for effective SARS-CoV-2 replication and acts as a proviral host factor for SARS-CoV-2.

### Pharmacological AhR inhibition limits the replication of SARS-CoV-2 and its variants

**Fig. 4.**
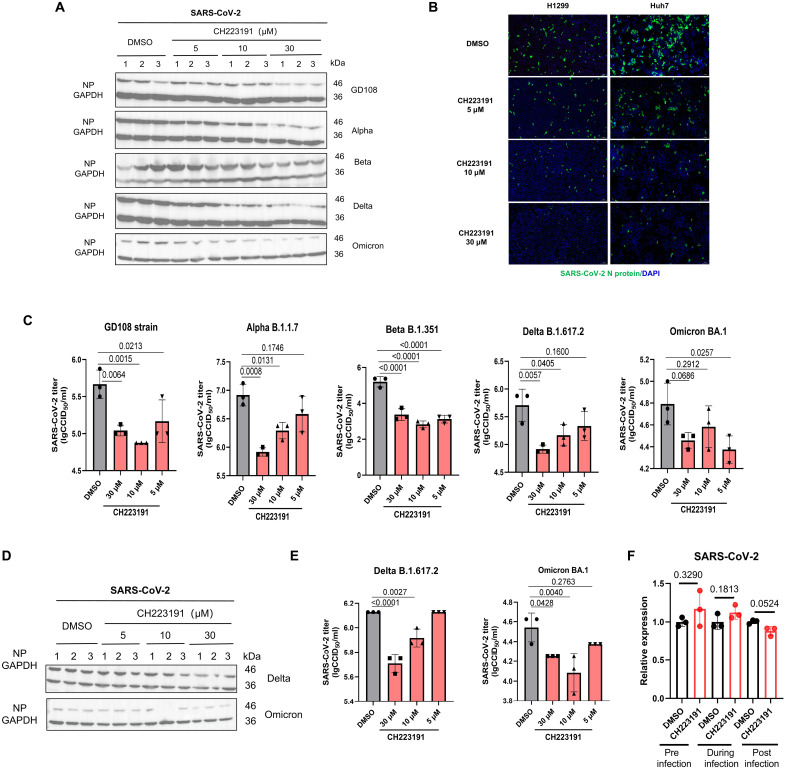
Pharmacological AhR inhibition limits replication of SARS-CoV-2 and its variants in vitro. (**A**) Immunoblot analysis of SARS-CoV-2 NP protein expression in CH223191- or DMSO-treated Huh7 cells. Huh7 cells were pretreated with different concentrations of CH223191 or DMSO and infected with SARS-CoV-2 (MOI of 0.1) for 24 hours. (**B**) Immunofluorescence assay of SARS-CoV-2 NP protein expression in CH223191- or DMSO-treated Huh7 cells by microscopy (scale bars, 200 μm). (**C**) Viral titer was determined by CCID_50_ assay in the supernatants of Huh7 cells pretreated with CH2213191 or DMSO and infected with SARS-CoV-2 (MOI of 0.1) for 24 hours. (**D**) Immunoblot analysis of SARS-CoV-2 NP protein expression in CH223191- or DMSO-treated Calu-3 cells. Calu-3 cells were pretreated with different concentrations of CH223191 or DMSO and infected with SARS-CoV-2 (MOI of 0. 05) for 24 hours. (**E**) Viral titer was determined by CCID_50_ assay in the supernatants of Calu-3 cells pretreated with CH2213191 or DMSO and infected with SARS-CoV-2 (MOI of 0.05) for 24 hours. Data are presented from at least three independent experiments (means ± SD). *P* values were determined using a two-tailed, unpaired Student’s *t* test. (**F**) qPCR quantification of SARS-CoV-2 RNA in Huh7 cells treated with CH223191 or DMSO preinfection, during adsorption or internalization of SARS-CoV-2 (MOI of 2). The cells were harvested at 2 hpi for qPCR to determine viral RNA level. Data from at least three independent experiments (means ± SD). *P* values were determined by using a two-tailed, unpaired Student’s *t* test.

Because AhR is necessary for SARS-CoV-2 replication, we evaluated the regulatory effect of AhR on the viral replication of SARS-CoV-2 and its variants using the AhR antagonist CH223191 (*27*) and found that AhR pharmacological inhibition limited SARS-CoV-2 replication. Small-molecule agonist and antagonist commonly have off-target effects. Therefore, to verify that the effects on SARS-CoV-2 infection observed throughout this study are due to AhR signaling perturbation, we first confirmed the specificity of the agonist and antagonist on AhR signaling. We detected the expression level of *AhR*, *CYP1A1*, and *CYP1B1* genes by qPCR after treatment of I3S or CH223191 in H1299, HepG2, and Huh7 cells. Our results showed that AhR signaling could be specifically modulated by I3S or CH223191 (fig. S4). Significantly, the specific regulatory effect of I3S or CH223191 on AhR signaling was also confirmed in the study of Zika virus by Giovannoni *et al.* (*18*). Next, we observed that the N protein expression of prototype SARS-CoV-2 (GD108) and its Alpha, Beta, Delta, and Omicron variants were markedly inhibited at 24 hpi by CH223191 in Huh7 cells in a dose-dependent manner (Fig. 4A). Immunofluorescence analysis of the N protein showed that the fluorescence decreased gradually with the increase in CH223191 concentration in H1299 and Huh7 cells (Fig. 4B). The viral titers of SARS-CoV-2 prototype and its Alpha, Beta, Delta, and Omicron variants were also significantly decreased at 24 hpi in Huh7 cells when AhR was inhibited with CH223191 (Fig. 4C). Similar results were observed in human lung cancer cell line Calu-3 (Fig. 4, D and E). To study at which stage AhR inhibition interferes with the process of virus infection, we treated Huh7 cells with CH223191 at different stages of SARS-CoV-2 infection (pretreatment before virus entry, virus adsorption period, and virus internalization period) and quantified the viral RNA by qPCR at 2 hpi. The results show that CH223191 had no effect on SARS-CoV-2 replication at the early stage of infection, suggesting that the promotion of AhR on SARS-CoV-2 replication occurs in the late stage of infection (Fig. 4F). Notably, no cytotoxicity was detected for CH223191- and I3S-regulated viral replication (fig. S5). These results suggested that the pharmacological inhibition of AhR limits the replication of SARS-CoV-2 and its variants in vitro. In conclusion, these findings suggested that AhR signaling promotes SARS-CoV-2 replication and acts as a host proviral factor, thereby deeming AhR as a potential pan-SARS-CoV-2 antiviral target against SARS-CoV-2 and its variants.

### AhR boosts SARS-CoV-2 replication by limiting IFN-I response and up-regulating ACE2 transcription

**Fig. 5.**
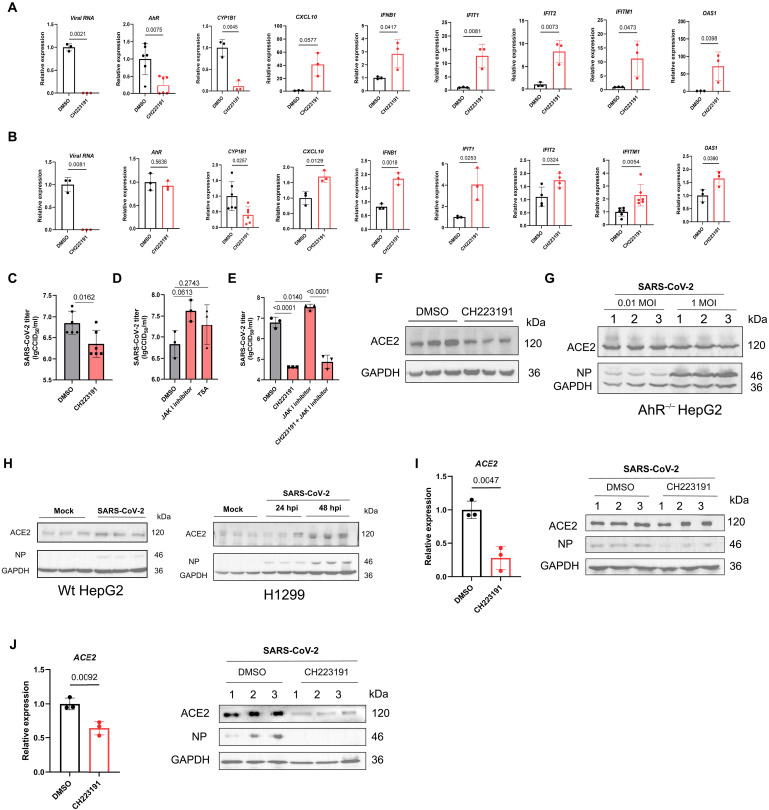
AhR boosts SARS-CoV-2 replication by limiting IFN-I response and up-regulating ACE2 transcription. Relative expression of cellular genes and viral RNA levels was determined by qPCR in SARS-CoV-2–infected (MOI of 0.1) H1299 cells (**A**) or HepG2 cells (**B**) pretreated with CH223191 or DMSO. (**C**) Viral titer was determined by CCID_50_ assay in the supernatants of Vero cells pretreated with CH2213191 or DMSO and infected with SARS-CoV-2 (MOI of 0.05) for 24 hours. (**D**) Effect of inhibitors of IFN signaling on SARS-CoV-2 replication determined 24 hpi by CCID_50_ assay. HepG2 cells pretreated with DMSO, Janus kinase 1 (JAK1) inhibitor, or trichostatin A (TSA) and infected with SARS-CoV-2 (MOI of 0.1), and, 24 hpi, supernatants were harvested for CCID_50_ assay. (**E**) Effect of CH223191 on HepG2 cells treated with JAK1 inhibitor. HepG2 cells were treated with JAK1 inhibitor and CH223191 as indicated and infected with SARS-CoV-2 (MOI of 0.1), and, 24 hpi, supernatants were harvested for CCID_50_ assay. (**F**) Immunoblot analysis of ACE2 protein in HepG2 cells treated with CH223191 for 12 hours. (**G**) Immunoblot analysis of ACE2 and SARS-CoV-2 NP protein in AhR^−/−^ HepG2 cells infected with SARS-CoV-2 (MOI of 0.01 or 1) for 24 hours. (**H**) Immunoblot analysis of ACE2 and SARS-CoV-2 NP protein in H1299 or HepG2 cells infected with SARS-CoV-2 (MOI of 0.1) for 24 and 48 hours. (**I**) Effect of CH223191 on ACE2 mRNA and protein or SARS-CoV-2 NP protein in HepG2 cells or (**J**) H1299 cells. HepG2 or H1299 cells were pretreated with CH223191 and infected with SARS-CoV-2 (MOI of 0.1), and, 24 hpi, RNA and protein were harvested for qPCR and immunoblot analysis, respectively.

As a nuclear receptor and transcription factor, AhR inhibits the expression of IFN-I (*28*). IFN-I is known to inhibit the replication of the SARS-CoV-2 through ISGs (*29*, *30*). Thus, this coincidence prompted us to hypothesize that SARS-CoV-2 activates AhR to promote viral replication by suppressing IFN-I in SARS-CoV-2–infected hosts. On the basis of this speculation, we studied the role of AhR in the control of the antiviral response during SARS-CoV-2 replication. The potential effects of AhR on the expression of IFN and ISGs were investigated using human lung epithelial cell line H1299 and human liver cancer cell line HepG2, which express ACE2 and can be infected by SARS-CoV-2. The inhibition of AhR increased the expression of IFN-I and ISGs in H1299 cells infected with SARS-CoV-2 but decreased the SARS-CoV-2 viral RNA level (Fig. 5A). In human HepG2-infected with SARS-CoV-2, similar results were observed after inhibition of AhR by CH223191 (Fig. 5B). These findings suggested that AhR promotes SARS-CoV-2 replication by limiting IFN-I–mediated host antiviral response. AhR antagonists limit AhR activation and enhance the host antiviral response, thereby reducing viral replication. Next, we determined whether AhR-promoted virus replication depends on the inhibition of IFN-I. Vero cells that have normal AhR expression but deficient IFN expression as a result of spontaneous gene deletions were used. The cells treated with CH223191 at 24 hpi showed a decreased SARS-CoV-2 replication by about 0.5 lgCCID_50_/ml compared to dimethyl sulfoxide (DMSO) treatment (Fig. 5C). Consistent with these results, the viral replication was also increased in the HepG2 cells infected with SARS-CoV-2 and containing IFN-I signalinginhibiting drugs, such as Janus kinase 1 (JAK1) inhibitor and trichostatin A (TSA) (Fig. 5D). Thus, these findings suggested that the activation of AhR triggered by SARS-CoV-2 inhibits IFN-I–dependent response, thus promoting SARS-CoV-2 replication. In addition, virus replication was restricted by IFN-I–independent mechanisms (*31*, *32*). Thus, it could be deduced that AhR inhibition can reduce SARS-CoV-2 infection in IFN-I–deficient Vero cells, which was further confirmed by quantification of virus production (Fig. 5D). In addition, AhR inhibition attenuated viral replication when IFN-I signaling was inhibited with Jak1 inhibitor in HepG2 cells (Fig. 5E). Overall, these results suggested that AhR also interferes with IFN-I–independent antiviral mechanisms to promote SARS-CoV-2 replication. We found that the expression of ACE2, a cellular receptor of SARS-CoV-2 that mediated viral entry, was affected after treatment with CH223191 or I3S (Fig. 5F and fig. S4), suggesting that the expression of ACE2 was regulated by AhR signaling. We further observed that the expression of ACE2 was not affected in AhR^−/−^ HepG2 cells infected with SARS-CoV-2 (Fig. 5G). By contrast, the expression of ACE2 was up-regulated in H1299 and HepG2 cells during SARS-CoV-2 infection (Fig. 5H) but decreased when AhR was inhibited with CH223191 in both cell lines, as determined by RT-qPCR and Western blotting (Fig. 5, I and J). Together, these findings suggested that AhR regulates the expression of ACE2. Mechanistically, the transcription factor AhR binds to the promoter of *ACE2* gene, as proven by chromatin immunoprecipitation (ChIP)–qPCR from Lv *et al.* (*33*). The study showed that AhR directly regulates *ACE2* transcription and hence is crucial for SARS-CoV-2 cell entry (*33*). Our results also confirmed the transcriptional regulation effect of ACE2 by AhR. Because ACE2 mediates SARS-CoV-2 infection of alveolar epithelial cells, the up-regulation of ACE2 expression is expected to prompt SARS-CoV-2 infection and aggravate lung pathology. Collectively, these findings indicated that AhR boosts SARS-CoV-2 infection via limiting IFN-I–dependent antiviral mechanisms and up-regulating the expression of ACE2 receptor.

### AhR inhibition reduces SARS-CoV-2 replication and ameliorates pneumonia inflammation in hamsters

**Fig. 6.**
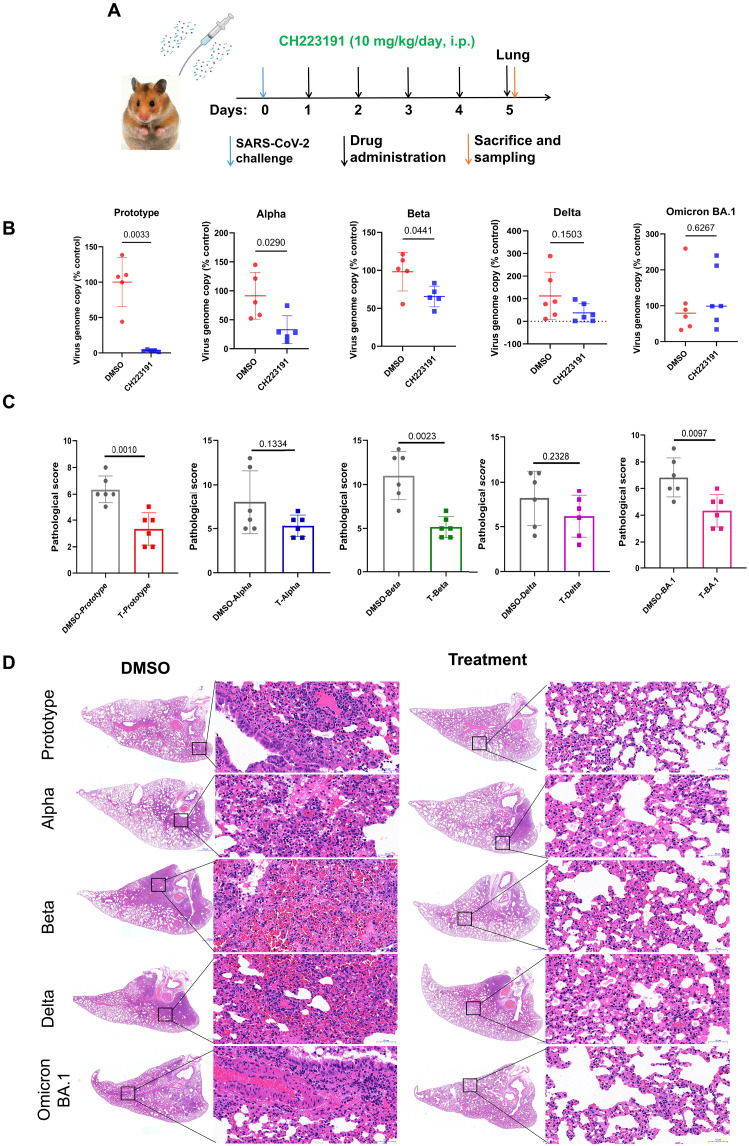
AhR antagonist limits SARS-CoV-2 replication and ameliorates pneumonia inflammation in hamsters. (**A**) Schematic of SARS-CoV-2 infection and animal operations. Hamsters were infected with SARS-CoV-2 and treated with control DMSO or CH223191 10 mg/kg, intraperitoneally (i.p.) for 5 days. (**B**) Relative viral load of SARS-CoV-2. The hamster lung tissues were used for quantifying viral load by real-time RT-PCR. (**C**) Comprehensive pathological scores for lung sections. The data represent means ± SD. *P* values were determined by using a two-tailed, unpaired Student’s *t* test. (**D**) Representative lung histopathological images [hematoxylin and eosin (H&E) staining] for lung lobe sections collected from SARS-CoV-2–infected hamsters at 5 days postinfection (dpi). Scale bars, 50 μm.

**Fig. 7.**
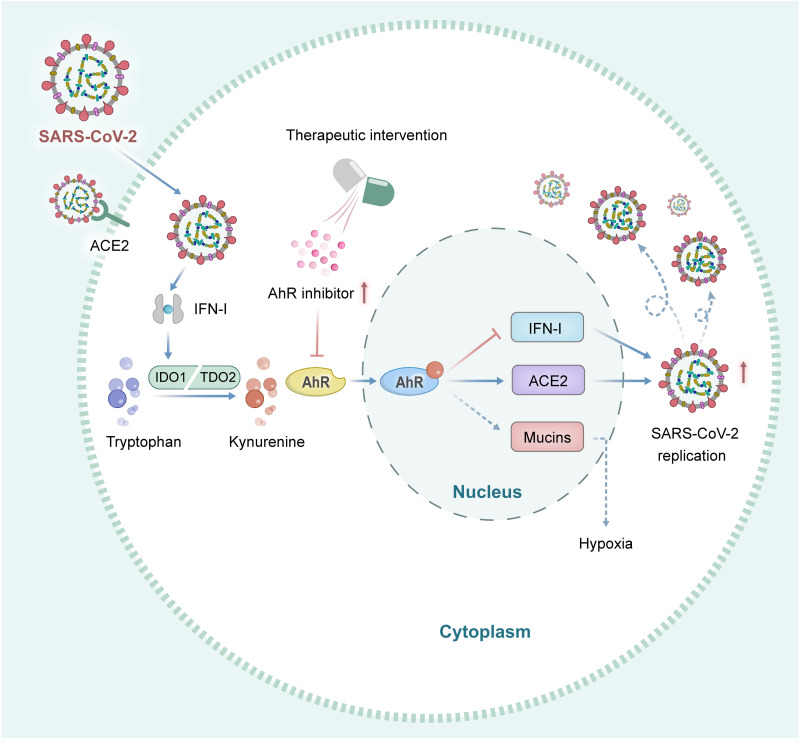
Schematic diagram of AhR promoting SARS-CoV-2 replication and acting as a therapeutic target. AhR was activated by SARS-CoV-2 infection by Trp-Kyn metabolism pathway. The activated AhR inhibits the host antiviral responses mediated by IFN-I and up-regulates ACE2 expression, thus promoting viral replication. AhR antagonists block the activation of AhR, enhance the host antiviral response, and reduce the expression of ACE2 receptor, thus reducing viral replication.

On the basis of our findings on the role of AhR as a proviral host factor for SARS-CoV-2 replication, we hypothesized that the pathological damage caused by the virus infection in the lungs could be alleviated by inhibiting AhR activity. Hence, the therapeutic potential of AhR antagonist CH223191 was evaluated in Syrian hamsters infected with SARS-CoV-2 and its variants. Syrian hamsters are highly sensitive to SARS-CoV-2 and can be used as a SARS-CoV-2 infection model to evaluate the drug and vaccine effects (*34*). First, the animals were infected with 10^3^ CCID_50_ of SARS-CoV-2 or its variants and then injected with the AhR inhibitor CH223191 intraperitoneally (10 mg/kg body weight) consecutively for 5 days (Fig. 6A). Subsequently, the hamsters were euthanized, and the lung tissues were analyzed. As expected, the viral load was markedly decreased in the CH223191-treated groups for the prototype, Alpha, Beta, and Delta variants of SARS-CoV-2, except for Omicron variant with a basically unchanged trend, as shown by RT-qPCR results (Fig. 6B). A decline of >50% in the relative viral load was observed in CH223191-treated hamsters compared to the control DMSO-treated group, indicating that AhR inhibition by CH223191 limits SARS-CoV-2 and its variants replication in vivo. Furthermore, to more robustly characterize the impact of CH223191 in vivo, we further detected the expression level of ACE2 and ISGs after treatment in vivo. Our results showed that the expression level of ACE2 in CH223191-treated group was significantly decreased compared with DMSO-treated group. In addition, the expression levels of ISGs in CH223191-treated group were significantly increased compared with DMSO-treated group (fig. S6). These results indicated that AhR inhibition reduces ACE2 expression and promoted ISGs expression. Moreover, the pathological damage induced by SARS-CoV-2 or its different variants was ameliorated in the infected hamsters’ lungs after CH223191 treatment compared to the control groups (Fig. 6, C and D), as shown by reduced inflammation, less alveolar hemorrhage, thinner alveolar walls, and lesser inflammatory cell infiltration. This phenomenon suggested that AhR antagonists ameliorate lung pathology partially by limiting viral replication. In addition, body weight was monitored within 5 days postinfection (dpi). CH223191 exerted a slight protection against infection-associated weight loss of hamsters caused by SARS-CoV-2 infection (fig. S7). Together, these in vivo data strongly supported a role for AhR signaling in SARS-CoV-2 replication and pathogenesis and identified AhR antagonism as a candidate therapeutic approach for COVID-19 (Fig. 7).

## DISCUSSION

The persistent COVID-19 pandemic, the periodic recurrence of virus infection, and challenging variants have necessitated the development of novel broad-spectrum antiviral therapies. Targeting the universal host factors required for virus replication rather than viral enzymes is the most promising approach, with the advantages of broad spectrum and low drug resistance (*12*, *35*). However, despite recent reports that the transcriptional regulator BRD2 and a host cysteine-aspartic protease caspase-6 are involved in SARS-CoV-2 replication (*36*, *37*), little is known about other host factors or how to target them therapeutically as antiviral targets. In this study, we reported that Kyn production triggered by SARS-CoV-2 leads to AhR activation, inhibition of IFN-I expression, and the transcriptional activation of ACE2, indicating that AhR is a proviral host factor for SARS-CoV-2 replication, similar to previous findings with respect to other viral infections (*16*, *18*, *19*). The current study elucidated the mechanism by which AhR promotes SARS-CoV-2 replication with respect to inhibition of antiviral innate immunity and activation of viral ACE2 receptor.

IFN-I is a central regulator of antiviral response that induces the expression of AhR; conversely, AhR inhibits the expression of IFN-I, indicating that AhR negative feedback regulates immunity. It also limits the potentially deleterious activity of innate immune cells and effector T cells (*38*) and inhibits another key effector molecule nuclear factor κB in host antiviral and inflammatory responses (*18*, *28*, *39*, *40*). Therefore, these findings suggested that SARS-CoV-2 uses AhR-driven immune regulatory mechanism to evade immune response, similar to the recent report on AhR activation in tumor immune evasion (*41*). Previous studies have shown that the replication of influenza A, Zika, and dengue viruses is reduced after inactivation of AhR with AhR antagonists and gene knockout methods (*18*, *19*). Notably, AhR antagonists increased the IFN-β levels in mice infected with influenza A virus. Moreover, the virus titer in BALF was reduced, and the survival rate of the animals was improved (*19*). In the preclinical mouse model, AhR antagonists also reduced the replication of Zika virus in the fetus and improved the congenital Zika virus syndrome (*18*). These findings suggested that AhR is a proviral host factor targeted by several viruses that limits IFN-I–driven host antiviral immunity and enhances virus replication. The identification of AhR as a proviral host factor has therapeutic significance for infectious viral diseases.

In the current study, AhR inhibitor increased IFN-I signaling, down-regulated ACE2 expression, and reduced viral load and lung pathology. The antiviral drugs targeting host factors provide an alternative approach to traditional antiviral drugs targeting viral proteins. Coincidentally, a recent study also reported a strategy for the broad-spectrum prevention and treatment of COVID-19 by targeting protease TMPRSS2, involved in viral entry (*42*). However, this strategy might have potential side effects resulting from the targeting of the host cell pathway. The development of AhR-targeted therapy should consider the ligand and cell-specific effects of AhR signaling and how these effects are regulated by tissue microenvironment. AhR agonists are not only produced by host metabolism but also provided by environmental pollutants, diet, and the commensal flora (*43*). A wide range of sources for this agonist provides clues for the AhR-mediated mechanisms by which individuals in the population exhibit heterogeneous susceptibility to SARS-CoV-2 infection and virus-induced pathology.

In the past decade, AhR has become a key regulator of the immune system. Most immune cells express AhR constitutively or after induction. Previous studies confirmed that AhR is the main regulator of mucosal barrier function (*44*, *45*). The importance of AhR-mediated response after activation of endogenous and exogenous ligands indicated that the respiratory system is sensitive to changes in AhR expression or function. Some studies have also confirmed that AhR regulates the immune response of various respiratory diseases and shown that the lung is sensitive to ligands using the gene-deficient mice that were given the agonists and antagonists (*44*, *46*–*48*). In addition, our results suggested that the pharmacological inhibition of AhR ameliorates lung inflammation because it inhibits the replication of SARS-CoV-2, intercellular adhesion, mucin production, and cytokine expression in the lungs. A recent study by Liu *et al.* (*20*) reported that SARS-CoV-2–triggered IFN-AhR signaling induces mucin overproduction in lung epithelial cells, thus triggering COVID-19–related hypoxia. The study also showed that the pharmacological inhibition of AhR during SARSCoV-2 infection reversed the virus-induced lung pathology. However, the effects of AhR inhibition on lung SARS-CoV-2 replication were not assessed. Hence, the reduction in virus-induced lung pathology might result from the suppression of SARS-CoV-2 replication and not only due to decreased mucin production. Therefore, the present study aimed to evaluate the effect of AhR inhibition on SARS-CoV-2 replication and proved that the pharmacological AhR inhibition limits the replication of SARS-CoV-2 and its variants by promoting IFN-I response and down-regulating the transcription of ACE2. Therefore, we can conclude that AhR antagonists ameliorate lung pathology by enhancing antiviral immunity, limiting viral replication, and inhibiting excessive mucus production. Overall, on the basis of the reported role of AhR in the control of antiviral immunity, these findings supported the use of AhR antagonists to treat SARS-CoV-2 and its variants. Broad-spectrum antivirals are clinically needed for the effective control of SARS-CoV-2 and its variants. However, although significant efforts have been made to discover the therapeutic antiviral agents for coping with emerging SARS-CoV-2 and its variants, specific and effective drugs with low toxicity have been rarely reported. Our in vivo data on pan-SARS-CoV-2 effectiveness of the antiviral therapy of AhR used as a target provide a new strategy for the development of broad-spectrum antivirals. Significantly, a recent study also confirmed that the nuclear receptor farnesoid X receptor inhibition may protect from SARS-CoV-2 infection by reducing ACE2 (*49*). This further suggested the feasibility of nuclear receptor AhR as a pan-SARS-CoV-2 therapeutic target.

In summary, we showed that SARS-CoV-2 infection activates AhR signaling and facilitates viral replication by interfering with IFN-I–driven antiviral immunity and up-regulating ACE2 receptor expression (Fig. 7). Furthermore, pharmacologic AhR blockade or knockout reduces the replication of SARS-CoV-2 and its variants in vitro. Drug targeting of AhR with AHR antagonists markedly reduced SARS-CoV-2 replication and reversed the pathological damage of lung inflammation caused by virus infection in hamsters. Together, AhR has been identified as a SARS-CoV-2 proviral host factor and a candidate host-directed broad-spectrum antiviral target against SARS-CoV-2 and its variants, including Omicron, and other potential variants in the future.

## MATERIALS AND METHODS

### Ethics and biosafety statement

All animal and cell infections of SARS-CoV-2 were conducted in the high-level biosafety facility of the National Kunming High-Level Biosafety Primate Research Center (Yunnan, China) according to the standard operating procedures of the biosafety level 3 and biosafety level 4 animal facilities. All animal experiments were approved by the Institutional Animal Care and Use Committee of Institute of Medical Biology, Chinese Academy of Medical Science (ethics number DWSP202207010).

### Cells, viruses, and reagents

Human non–small cell lung cancer cell line H1299, human bronchial epithelial cell line BEAS-2B, human lung adenocarcinoma cell line Calu-3, and human hepatoma cell lines HepG2 and Huh7 were purchased from Procell Life Science and Technology Co. Ltd. (Wuhan, China) and cultured in RPMI 1640 medium (Gibco, USA) or Dulbecco’s modified Eagle’s medium (DMEM) (Gibco) with 10% fetal bovine serum (FBS) and penicillin- streptomycin (100 U/ml). Vero cells (African green monkey kidney; CCL-81) were used to generate viral stocks and cultured in DMEM (Gibco) with 10% FBS and penicillin (100 U/ml) and streptomycin (100 μg/ml). The following virus strains were used in this study: the prototypic SARS-CoV-2 strain [GD108 from Guangdong Center for Disease Control and Prevention (CDC)], Beta variants (B.1.351, GDPCC-nCOV84, and CSTR.16698.06.NPRC 2.062100001 from Guangdong CDC), Alpha variants [B.1.1.7, SARS-CoV-2/C-Tan-BJ202101(B1.1.7), and CSTR.1669 8.06. NPRC 2.062100002 from China CDC], Delta variants (B.1.617.2, CQ79, and CSTR.16698.06. NPRC 6. CCPM-B-V-049-2105-8 from Chongqing CDC), and Omicron variant (B.1.1.529 CCPM-B-V-049-2112-18 from the Institute of Laboratory Animal Sciences, Chinese Academy of Medical Sciences and Peking Union Medical College). The viral titer was determined by a standard TCID_50_ method. Cells were infected at an MOI of 0.1 and incubated at 37°C for 1 hour; then, the viral inoculum was removed, and fresh medium with 2% FBS was supplemented. IFN-β was purchased from PeproTech (catalog no. 300-02 BC, USA). Small molecules include CH223191 (catalog no. 3858, Tocris; catalog no. S7711, Selleck) at 5 to 30 μM, I3S (lot no. BCCF7082, Sigma-Aldrich) at 50 to 600 μM, TSA (ready-made solution; catalog no. T1952, Sigma-Aldrich, USA), and JAK1 inhibitor (catalog no. SC-204021A, Santa Cruz Biotechnology, USA) at 1 μM. I3S and CH223191 were solubilized in DMSO (Sigma-Aldrich) to prepare the stock solution according to the manufacturer’s instructions. In addition, the cells were pretreated with CH223191 or I3S overnight before further infection.

### scRNA-seq analysis

Rhesus monkeys (3 to 5 kg, 3 to 5 years old) were used for this study. One monkey (no. 16001) was infected by intragastric administration of 1 × 10^7^ plaque forming units (PFU) of SARS-CoV-2 in 1 ml of phosphate-buffered saline (PBS), and another monkey (no. 16149) was intranasally injected with 1 × 10^6^ PFU of SARS-CoV-2 in 200 μl of PBS. One monkey (no. 16271) was intranasally and intragastrically treated with PBS as a control. Here, we analyzed the tissues collected on 4 dpi following intranasal and intragastrical inoculation. Single-cell suspension (300 to 600 living cells/μl determined by Countstar) was loaded onto the chromium single-cell controller (10x Genomics) to generate single-cell gel beads in the emulsion using the single-cell 3 Library and Gel Bead Kit V3 (10× Genomics, 1000075) and Chromium Single Cell B Chip Kit (10× Genomics, 1000074). scRNA-seq libraries were constructed and analyzed according to the method used in our previous study (*49*). The other clustering method was Seurat 3.0 (R package). Cells with <200 genes or if the gene number ranked in the top 1% or the mitochondrial gene ratio was >25% were regarded as abnormal and filtered out. Dimensionality reduction was performed using principal components analysis and visualized by *T*-distributed stochastic neighbor embedding (tSNE) and the Uniform Manifold Approximation and Projection (UMAP). The cell type was annotated by singleR (https://bioconductor.org/packages/devel/bioc/html/SingleR.html). An unbiased cell type was identified from scRNA-seq data by leveraging the reference transcriptomic datasets of pure cell types to infer the origin of each cell independently.

### Cell viability assay

Cells (5 × 10^4^ cells per well) cultured in 96-well plates were exposed to three concentration gradients of CH223191 or I3S for 24 hours, five wells for each concentration. Then, cell viability was measured by the CellTiter AQueous One Solution Cell Proliferation Assay (catalog no. G3582, Promega, USA), following the manufacturer’s instructions. The absorbance of each well was measured on the SpectraMax i3 Multi-Mode Microplate Reader (Molecular Devices).

### RNA extraction and RT-qPCR

Total RNA was extracted from cells using TRIzol (Invitrogen) and reverse-transcribed into complementary DNA with the GoScript Reverse Transcription System (catalog no. A5001, Promega). The PCR amplification was conducted on a CFX-96 real-time RT-PCR detection system (Bio-Rad) with the Go Taq qPCR Master Mix (catalog no. A6001, Promega). Sequences of all real-time PCR primers are listed in table S2. The real-time PCR data of the target genes were normalized to the expression of the reference gene *GAPDH* to compute relative gene expression, as described previously (*37*).

### Generation of AhR-deficient HepG2 cells using Cas9-sgRNA

AhR-deficient HepG2 cell line was generated by CRISPR-Cas9–mediated genome engineering (HanBio Company, Shanghai, China). Briefly, AhR–single-guide RNA (sgRNA) was ligated to pHBLV-U6-gRNA-EF1-CAS9-PURO lentivirus (HanBio Ltd. Shanghai, China), named as HBLV-h-h-AhR-Cas9-gRNA-PURO. Then, HepG2 cells were infected with HBLV-h-h-AhR-Cas9-gRNA-PURO lentivirus and treated with puromycin to screen the cells successfully infected with the virus. The genome DNA of the screened cell lines was extracted and analyzed by Sanger sequencing. In addition, genomic DNA was isolated from AhR knockout colonies and subjected to PCR and restriction enzyme digestion. Subsequently, the knockout effect was verified by DNA sequencing of the target region. The guide RNA sequences used were as follows: AhR gRNA, 5′-TCAAGTCAAATCCTTCCAAG-3′.

### Immunofluorescence

For immunofluorescence staining assay, 1 × 10^5^ cells per well were seeded in a 24-well plate consisting of sterilized coverslips and incubated overnight. The culture medium was replaced with serum-free medium, followed by infection with SARS-CoV-2 variants at an MOI of 0.05 for 1 hour. Subsequently, the viral inoculum was removed and replaced with fresh culture medium containing 2% FBS for 48 hours. After treatment, the cells were rinsed briefly in PBS, fixed using 4% paraformaldehyde for 24 hours permeabilized with 0.5% phosphate buffer solution and Tween-20 (PBST) for 30 min, blocked with 3% normal goat serum (0.1% PBST), and incubated with anti-AhR (1:200; catalog no. GTX129013, GeneTex) or SARS-CoV-2 nucleoprotein (NP) (1:200; catalog no. 40143-MM08, Sino Biological) at 4°C overnight. Then, the cells were washed and incubated with secondary antibodies at room temperature for 1 hour (Alexa Fluor 594–conjugated goat anti-rabbit, 1:500; Invitrogen, A-11012; Alexa Fluor 488–conjugated goat anti-mouse, 1:500; Invitrogen, A-11001). Images were captured using a panoramic MIDI digital scanner (3DHISTECH, Hungary).

### Immunoblot analysis

Cells from one confluent well of a six-well plate were lysed in radioimmunoprecipitation assay lysis buffer (1×) supplemented with protease inhibitor cocktail (Cell Signaling Technology). Total cell lysate (15 to 20 μg) was separated by 4 to 20% bis-tris SDS–polyacrylamide gel electrophoresis and transferred to polyvinylidene difluoride membranes (Millipore). Then, the membranes were blocked in 5% nonfat dry milk and inoculated with the following antibodies overnight: anti-human AhR (catalog no. GTX129013, GeneTex), anti–SARS-CoV-2 NP (catalog no. MA5-36270, Invitrogen), anti–glyceraldehyde-3-phosphate dehydrogenase (catalog no. ab181602, Abcam), and anti-ACE2 (catalog no. ab108252, Abcam). Membranes washed four times with tris-buffered saline and 0.1% Tween 20 were incubated with horseradish peroxidase–conjugated secondary antibodies (goat anti-rabbit; catalog no. SA00001–2, Proteintech; goat anti-mouse; catalog no. SA00001-1, Proteintech) and developed using enhanced chemiluminescence substrate. The results were confirmed by at least three biological replicates.

### Estimation of Kyn and Trp concentrations by ELISA

Kyn and Trp levels in the cell supernatant were measured by enzyme-linked immunosorbent assay (ELISA) (catalog nos. K3728 and K3730, Immundiagnostik), according to the manufacturer’s instructions.

### Animal experiments and treatment protocol

Six- to eight-week-old male hamsters were purchased from Beijing Vital River Laboratory Animal Technology Co. Ltd. (Beijing, China). Animal experiments involving SARS-CoV-2 were carried out in an animal biosafety level 4 facility using positive pressure protective clothing. The procedures were approved by the Institutional Animal Care and Use Committee of the Institute of Medical Biology, Chinese Academy of Medical Sciences (DWSP202207010). The hamsters were also divided into treatment and control groups for each SARS-CoV-2 variant, containing six hamsters in each group. The hamsters were pretreated using AhR antagonist CH223191 (10 mg/kg, Selleck) or DMSO in advance 1 day before challenging with SARS-CoV-2. For SARS-CoV-2 infection, the hamsters were inoculated intranasally with SARS-CoV-2 at a dosage of 10^4^ TCID_50_ per hamster. After infection, the hamsters were treated with CH223191 or DMSO control once a day for 5 days, and their body weight and clinical symptoms were recorded. At 5 dpi, the hamsters were euthanized, and lung tissues were collected for real-time PCR and histopathological analysis. Tissues were homogenized, and the supernatants were used for RNA extraction.

### Histopathological analysis

Hematoxylin and eosin (H&E) staining was used to identify the histopathological changes in the lungs. Tissues of appropriate size were fixed in 10% neutral-buffered formalin for 4 days, embedded in paraffin, and sectioned (3 to 4 μm thick) for H&E staining. A pathologist blinded to the experiment scored semiquantitatively on the basis of alveolar wall thickening, intra-alveolar fibrin deposition (exudation), and inflammatory cell infiltration.

### Statistical analysis

All experiments contain at least three biological replicates. The results were expressed as means ± SD from three independent experiments. One-way analysis of variance (ANOVA) with Dunnett’s post hoc test was used for comparisons among multiple groups. The differences among two groups were compared using a two-tailed unpaired Student’s *t* test. The *P* value of <0.05 was considered a statistically significant difference. All statistical analyses were conducted using the GraphPad 9.0 software.

## Notes

View/request a protocol for this paper from *Bio-protocol*.
