# Supplementary material for: Aryl hydrocarbon receptor is a proviral host factor and a candidate pan-SARS-CoV-2 therapeutic target
Source: Sci Adv. 2023 May 31;9(22):eadf0211. doi: 10.1126/sciadv.adf0211 (PMC10413656; doi:10.1126/sciadv.adf0211)
Supplement: Supplementary file 1 — Figs. S1 to S7 Table S2 Legend for table S1 [file sciadv.adf0211_sm.pdf]

Science Advances
AAAS

Supplementary Materials for

Aryl hydrocarbon receptor is a proviral host factor and a candidate pan-SARS-CoV-2 therapeutic target

Jiandong Shi *et al.*

Corresponding author: Shuaiyao Lu, lushuaiyao-km@163.com; Yunzhang Hu, huyunzhangym@126.com; Xiaozhong Peng, pengxiaozhong@pumc.edu.cn

Sci. Adv. **9**, eadf0211 (2023)
DOI: 10.1126/sciadv.adf0211

The PDF file includes:

Figs. S1 to S7
Table S2
Legend for table S1

Other Supplementary Material for this manuscript includes the following:

Table S1

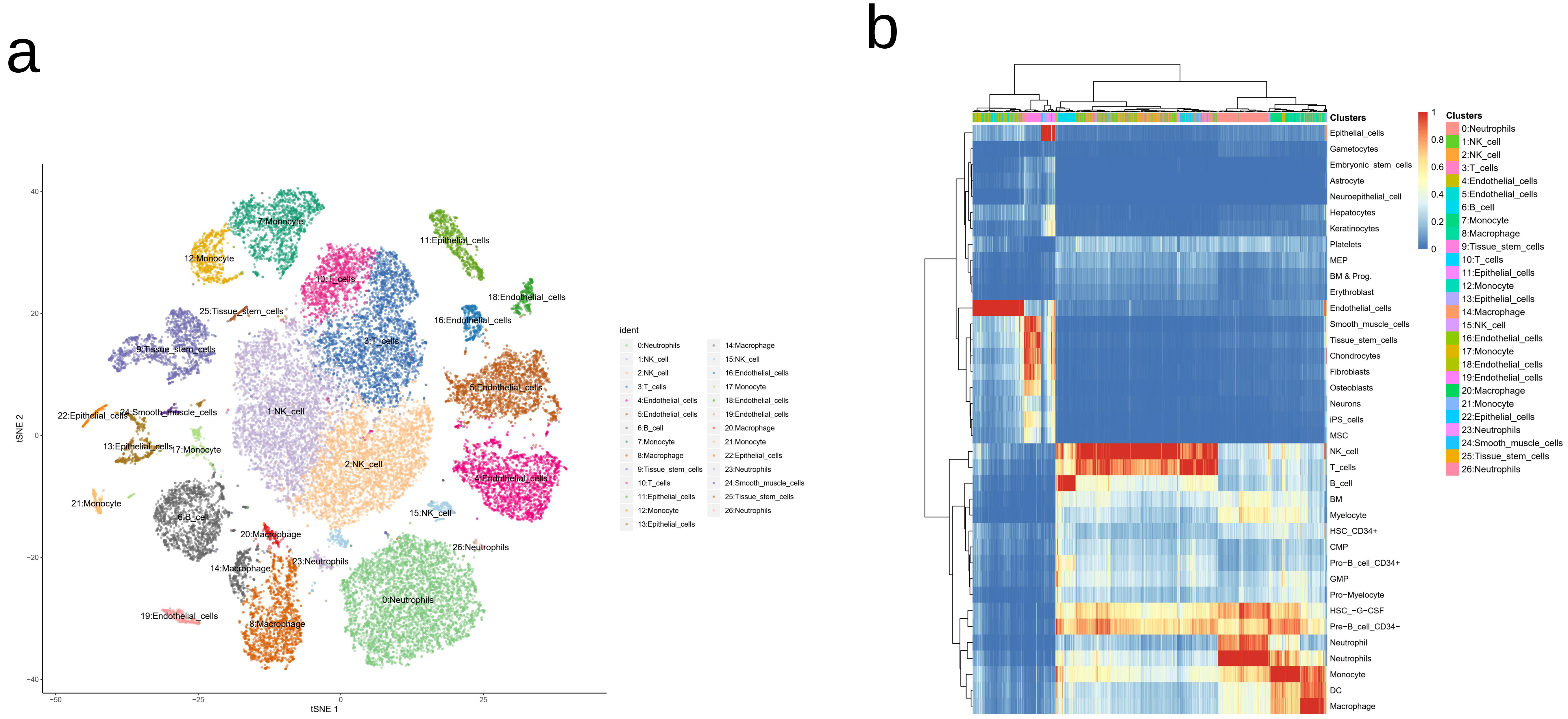

Fig. S1. tSNE projection of transcriptome from monkey lung tissue single cells.
(a) Clustering of single cells. Each dot corresponds to a single cell, colored according to subsets. **(b)** Heat map of single cell cluster score.

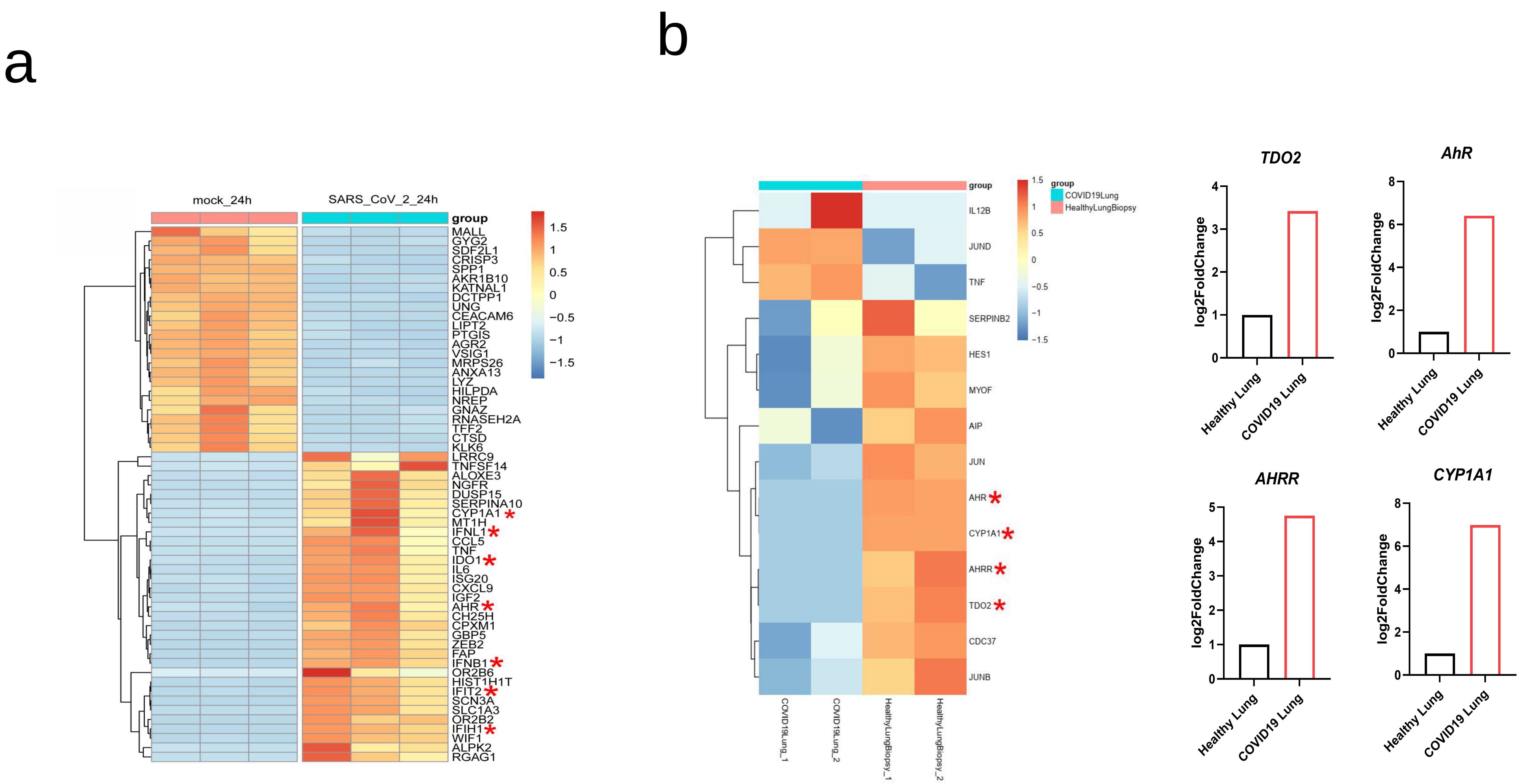

Fig. S2. AhR signaling is activated by SARS-CoV-2 infection in vitro and *in vivo*.
(a) Differentially expressed genes' analysis of RNA-seq data (https://bigd.big.ac.cn/; PRJCA00261) from mock-infected and SARS-CoV-2-infected (MOI=1, 24 hpi) Calu-3 cells. Red arrows indicate significantly upregulated genes in the AhR signaling pathway. **(b)** Differentially expressed genes' analysis of RNA-seq data (GEO accession GSE147507) from healthy and COVID-19 patients' lung biopsies. Red arrows indicate significantly upregulated genes in the AhR signaling pathway.

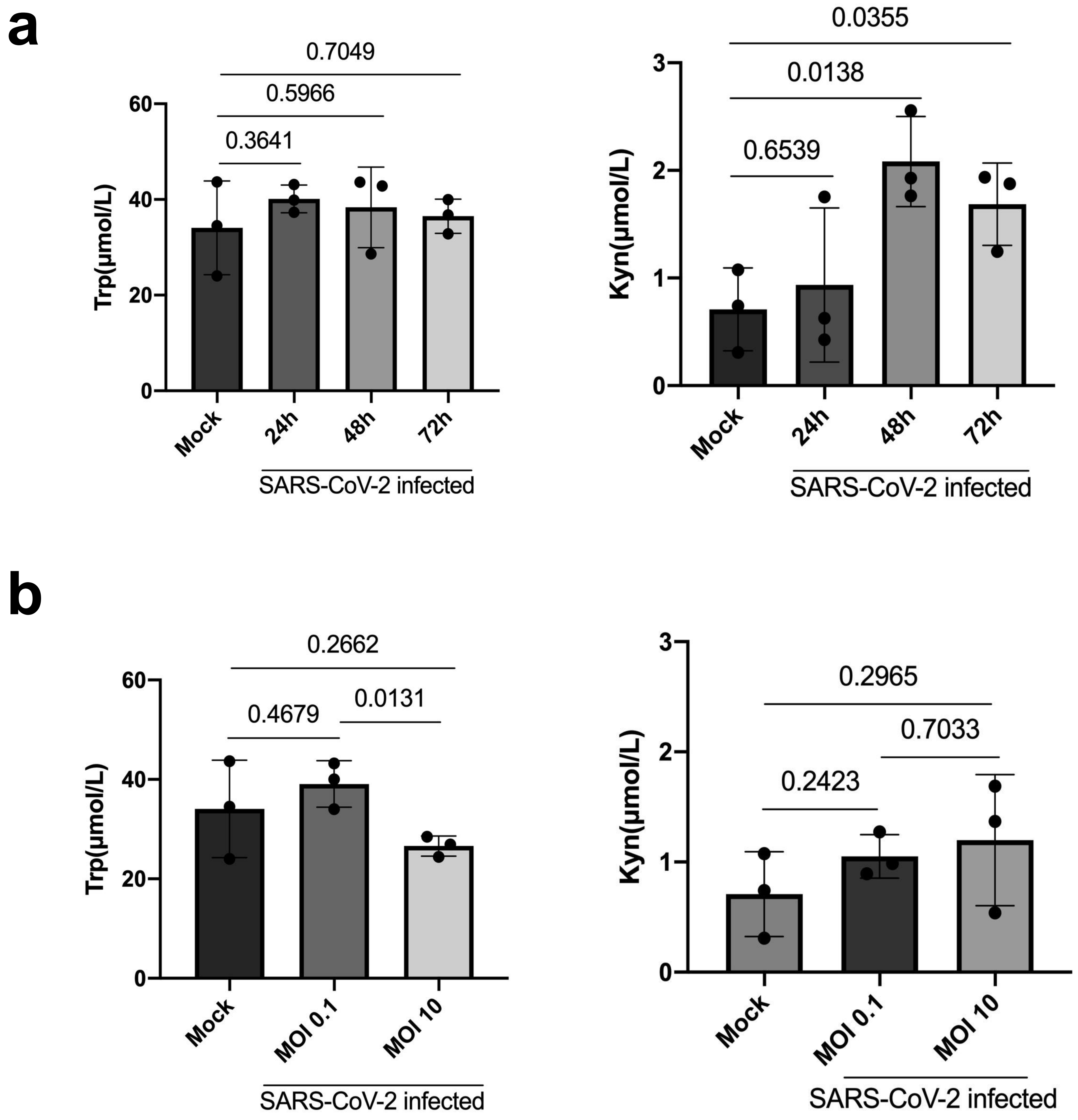

Fig. S3. ELISA quantification of Trp and Kyn in supernatants of SARS-CoV-2-infected BEAS-2B cells at different time points (a) with different MOIs (b). Data from at least three independent experiments (mean±SD). *P* values were determined using a one-way ANOVA followed by Tukey' s post hoc test.

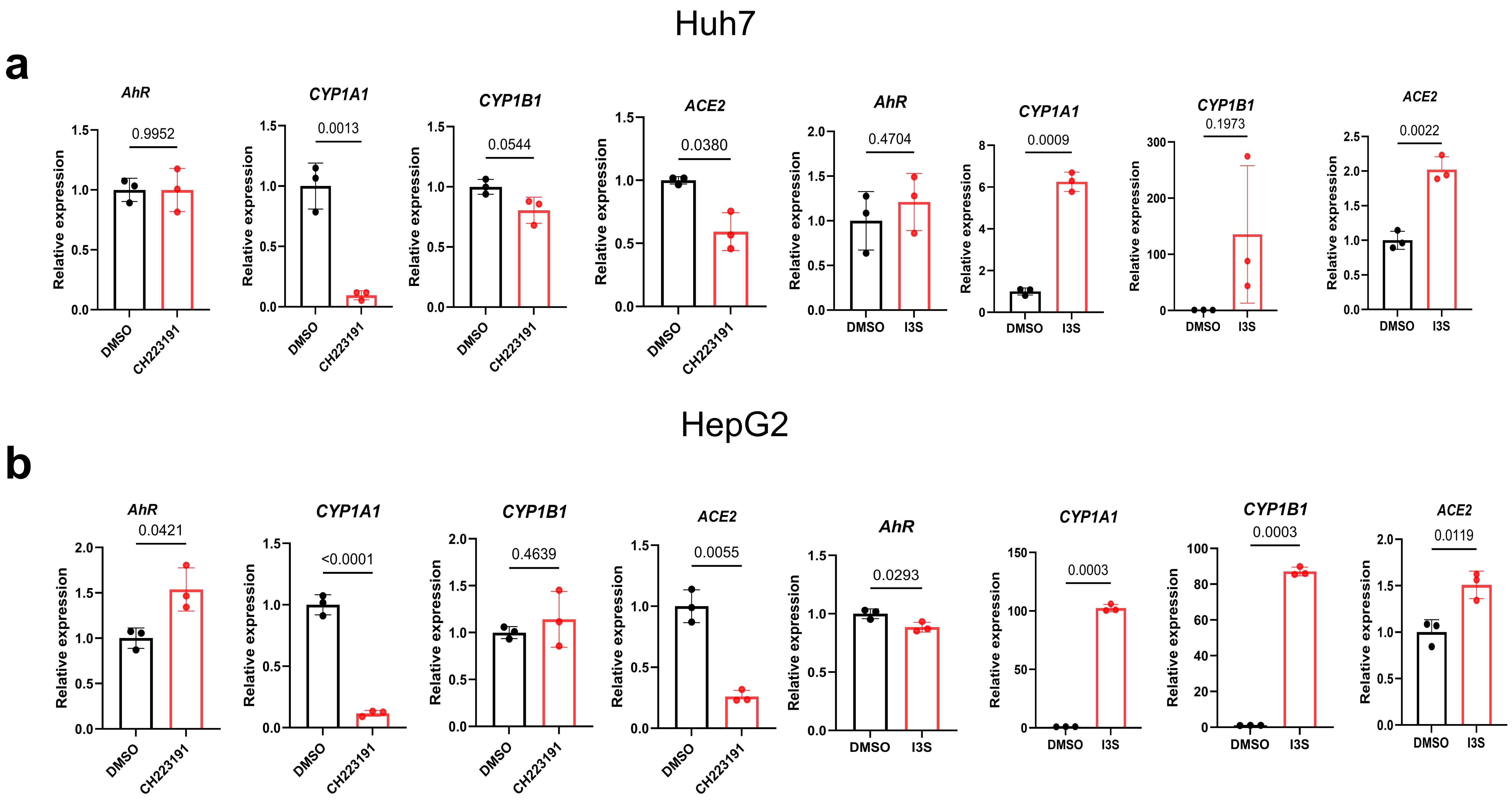

Fig. S4. AhR signaling modulation and ACE2 expression. Huh7 (a) and HepG2 cells (b) were treated with I3S or CH223191. 48h after treatment, cells were harvested forq PCRanalysisof AhR, CYP1A1, CYP1B1 and ACE2. Data represent the mean ± SD (n=3 independent experiments). *P* values were determined by a two-sided Student’ s t-test.

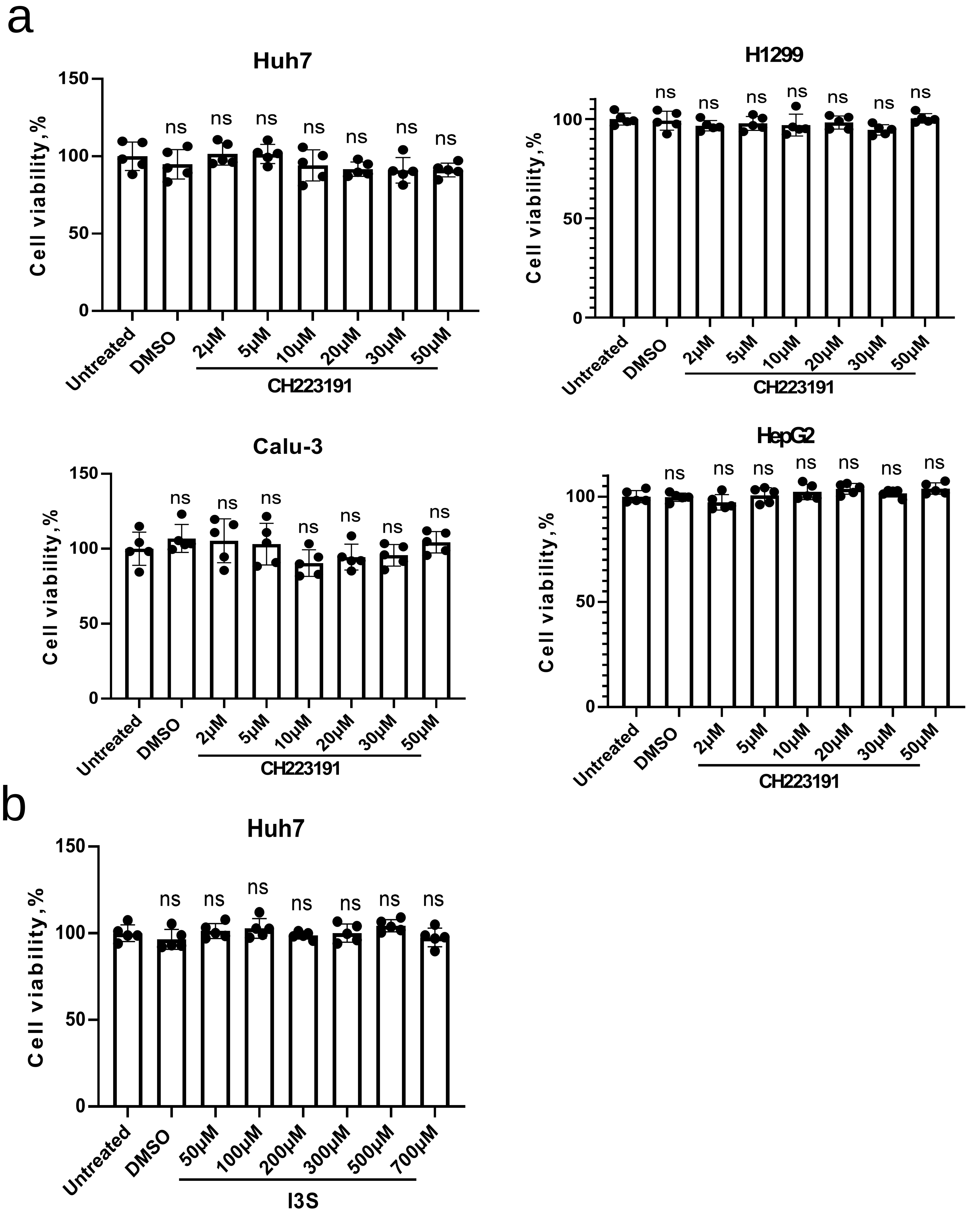

Fig. S5. Effect of CH223191 and I3S on cell viability and proliferation. (a) H1299, Huh7, Calu-3, and HepG2 cell viability after treatment with different concentrations of CH223191 for 24 h was evaluated by an MTS assay. Data represent the mean± SD (n=5 independent experiments). **(b)** Huh7 cells were incubated for 24 h with the indicated concentrations of I3S, and cell viability was evaluated by an MTS assay. Data represent the mean± SD (n=5 independent experiments). *P*-values were determined by one-way ANOVA, followed by Dunnett's post-hoc test. "ns" represents no significant difference.

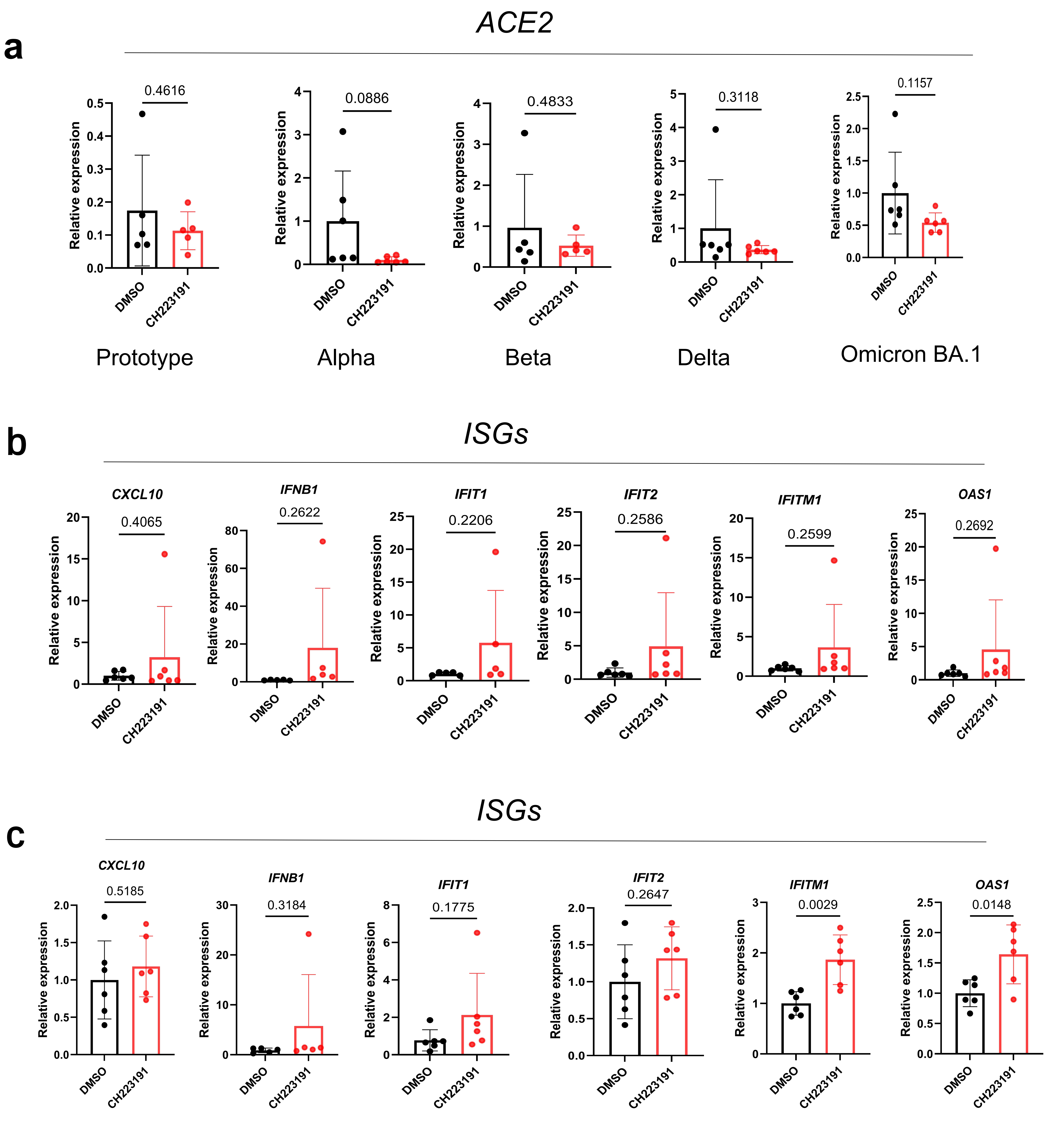

Fig. S6. Additional *in vivo* data of ACE2 and other ISG levels after pharmacological AhR inhibition. (a) ACE2 expression in groups infected with SARS-CoV-2 prototype and variants. **(b)** and (c) Interferon-stimulating genes (ISGs) levels in groups infected with SARS-CoV-2 prototype and Beta variant. *P* values were determined by a two-sided Student's t-test.

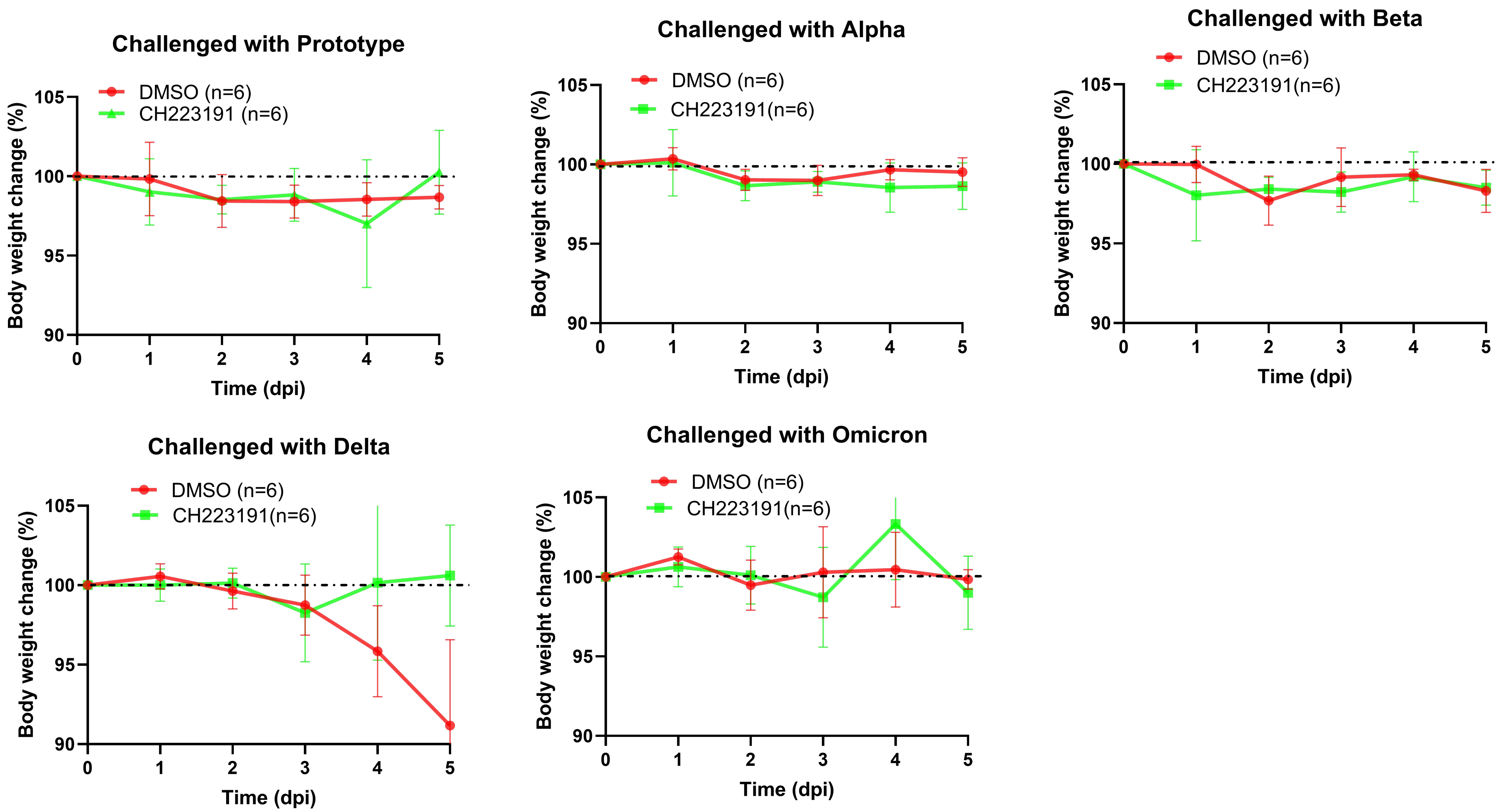

Fig. S7. Body weight changes in hamsters from 0 to 5 dpi (n=6). Body weight was observed daily.

Table S2. Primer sequences used in this study.

Gene	Primer name	Sequence (5' to 3')
IDO1	IDO1-F	GCCAGCTTCGAGAAAGAGTTG
	IDO1-R	ATCCCAGAACTAGACGTGCAA
TDO2	TDO2-F	TCCTCAGGCTATCACTACCTGC
	TDO2-R	ATCTTCGGTATCCAGTGTCGG
AhR	AhR-F	CAAATCCTTCCAAGCGGCATA
	AhR-R	CGCTGAGCCTAAGAACTGAAAG
CYP1A1	CYP1A1-F	ACATGCTGACCCTGGGAAAG
	CYP1A1-R	GGTGTGGAGCCAATTCGGAT
CYP1A2	CYP1A2-F	CTGGGCACTTCGACCCTTAC
	CYP1A2-R	TCTCATCGCTACTCTCAGGGA
CYP1B1	CYP1B1-F	AAGTTCTTGAGGCACTGCGAA
	CYP1B1-R	GGCCGGTACGTTCTCCAAAT
CXCL10	CXCL10-F	GTGGCATTCAAGGAGTACCTC
	CXCL10-R	TGATGGCCTTCGATTCTGGATT
IFNB1	IFNB1-F	GCTTGGATTCCTACAAAGAAGCA
	IFNB1-R	ATAGATGGTCAATGCGGCGTC
IFIT1	IFIT1-F	AGAAGCAGGCAATCACAGAAAA
	IFIT1-R	CTGAAACCGACCATAGTGGAAAT
IFIT2	IFIT2-F	GACACGGTTAAAGTGTGGAGG
	IFIT2-R	TCCAGACGGTAGCTTGCTATT
IFITM1	IFITM1-F	CCAAGGTCCACCGTGATTAAC
	IFITM1-R	ACCAGTTCAAGAAGAGGGTGTT
OAS1	OAS1-F	TGTCCAAGGTGGTAAAGGGTG
	OAS1-R	CCGGCGATTTAACTGATCCTG
ACE2	ACE2-F	ACAGTCCACACTTGCCCAAAT
	ACE2-R	TGAGAGCACTGAAGACCCATT
GAPDH	GAPDH-F	GGAGCGAGATCCCTCCAAAAT
	GAPDH-R	GGCTGTTGTCATACTTCTCATGG
SARS-CoV-2 N	N-F	GGGGAACTTCTCCTGCTAGAAT
	N-R	CAGACATTTTGCTCTCAAGCTG
SARS-CoV-2 ORF1ab	ORF1ab-F	CCCTGTGGGTTTTACACTTAA
	ORF1ab-R	GGGGAACTTCTCCTGCTAGAAT
Hamster γ-actin	Hamster γ-actin-F	ACAGAGAGAAGATGACGCAGATAATG
	Hamster γ-actin-R	GCCTGA ATGGCCACGTACA
Hamster *CXCL10*	Hamster CXCL10-F	GTGGGACTCAAGGAATCCCTC
	Hamster CXCL10-R	TGATGGCCTCAGACTCTGGATT
Hamster *IFNB1*	Hamster IFNB1-F	GCCAGACAAAGCAGAAGCAA
	Hamster IFNB1-R	TGATGAAGGCAGTGTCTCTC

Hamster *IFIT1*	Hamster IFIT1-F	GCCTATCGCCAGGATTTCAATGA
	Hamster IFIT1-R	TTCTGGATTTAACCTGACAGC
Hamster *IFIT2*	Hamster IFIT2-F	GAACACTTCATCCAGCAACA
	Hamster IFIT2-R	GCCTTCTCAAAACACACCTT
Hamster *IFITM1*	Hamster IFITM1-F	AACAACCACCATAATCAACAT
	Hamster IFITM1-R	CCCAGGCAGCAGAAGTTCAT
Hamster *OAS1*	Hamster OAS1-F	TCTCCAAGGTGGTGAAGGGCG
	Hamster OAS1-R	CCGTCGGTTTAACTGATCCTC
Hamster *ACE2*	Hamster ACE2-F	GGCTACAAGTACAGACTACAA
	Hamster ACE2-R	GCCATCTCATTTTTCAGGACC

Table S1. Differential expression of genes in rhesus monkey lung tissues in response to SARS-CoV-2 infection based on scRNA-seq.（Separate file）
